# Health Information Engagement Factors in Malaysia: A Content Analysis of Facebook Use by the Ministry of Health in 2016 and 2017

**DOI:** 10.3390/ijerph16040591

**Published:** 2019-02-18

**Authors:** Afiq Izzudin A. Rahim, Mohd Ismail Ibrahim, Faizul Nizam A. Salim, Mohd Ariff Ikram Ariffin

**Affiliations:** 1 Department of Community Medicine, School of Medical Science, Universiti Sains Malaysia, Kubang Kerian, Kota Bharu 16150, Kelantan, Malaysia; afiqizzudin@gmail.com; 2 Director General’s Office, Ministry of Health, Putrajaya 62590, Malaysia; drfaizulnizam@gmail.com (F.N.A.S.); mdariffikram@moh.gov.my (M.A.I.A.); 3 Corporate Communication Unit, Ministry of Health, Putrajaya 62590, Malaysia

**Keywords:** health information, social media, public health communication, health promotion

## Abstract

Health organizations have widely adopted social media for health promotion, public health communication conveyance, and organizational promotion activities. However, little published data exists on the factors that facilitate health information diffusion in South East Asia, especially Malaysia compared with Western countries. This study aimed to investigate factors associated with good engagement rates among internet users on the Facebook (FB) page of Ministry of Health Malaysia. In this observational study, 2123 FB posts were randomly selected. Data dated from 1 November 2016 to 31 October 2017 was gathered from the Facebook Insight. The logistic regression model was applied to identify factors associated with good engagement rates. This study found that a FB post with a good engagement rate was significantly associated with a health education post (Adjusted Odd Ratio (AOR): 3.80, 95% Confidence Interval CI: 3.02–4.78, *p* < 0.001), a risk communication post (AOR: 1.77, 95% CI: 1.39–2.26, *p* < 0.001), a post in the afternoon (AOR: 1.76, 95% CI: 1.34–2.31, *p* < 0.001) or in the evening (AOR: 1.48, 95% CI: 1.20–1.82, *p* < 0.001), and a video format (AOR: 3.74, 95% CI: 1.44–9.71, *p* = 0.007). Therefore, we present the first comprehensive analysis of health information engagement among internet users in Malaysia. The growing trends of online health information-seeking behaviors and demand for the availability of validated health information require effective strategies by public health organizations to disseminate health information and achieve better audience engagement on social media.

## 1. Introduction

Social media are a collection of digital channels and tools (e.g., Facebook, Twitter, Instagram, Sina Weibo, and YouTube) used increasingly for public health communication [1]. Social media, particularly Facebook, are widely utilized by health organizations worldwide and are fast becoming principal instruments of alternative communication channels to deliver health messages, conduct disease surveillance, spread health awareness, and address public health issues to the public [2,3,4,5]. Facebook (FB) has emerged as a powerful platform with several advantages compared with more traditional communication channels, as it has proven to be a cost-effective tool for the dissemination of health messages, capable of reaching hidden minorities for better public health interventions [6,7,8]. In recent years, there has been increasing public interest in Malaysia in searching for online health information related to their health problems. Evidence from local studies has shown that the public perceive the information provided to be useful and reliable, a perception that leads them to consult with any healthcare practitioner about their health conditions directly through social media [9,10,11]. Growing online health-seeking behaviors and increasing numbers of non-authorized health websites or social media accounts that share distorted health information with conflicts of interest (selling unproven alternative medical products and unregistered private health services) have made it necessary for health organizations to engage with internet users on social media effectively and strategically [1,4,10].

In the new digital era, engagement has become an indicator of mutual benefits received by health organizations and internet users [12]. The concept of social media engagement was the central focus among researchers and health promoters for enhancing the effectiveness of health information diffusion by health organizations through stimulation of interactive behaviors of internet users on social media. The engagement can be encouraged through viral reach (sharing), effective evaluation (liking), and message deliberation (commenting) [13,14]. These stimulations of interactive behaviors were generated by four key factors which are authority, privacy, evidence, and incentive appeals that influence the way people perceive health information [15]. Additionally, evidence suggested that technical aspects, such as types of health information, types of post, times of posts, marketing elements, and individual factors, including socio-demographic circumstances, Internet literacy, and educational background, were among the critical factors that influence Internet user engagement on social media [2,7,16]. In the context of this study, the interactions between the Ministry of Health (MOH), Malaysia and Facebook users were intended to promote better health knowledge through successful information dissemination and consumption.

As far as the present authors are aware, no study has previously been published to evaluate or analyze health information delivery or health communication engagement by any Malaysian health organization via social media. Most of the related studies were conducted in the origin country of social media giants, such as the U.S. and China, and other developed countries in Europe and the Oceania region [2,7,17,18,19,20]. A proper engagement analysis among internet users on social media will provide real-time feedback for government agencies and increase their accountability in decision-making regarding specific government policies [1,10]. Hence, our first objective is to determine the types of health information, types of posts, times of posts, and rates of good or poor engagement with posts on the MOH FB page. Our next objective is to determine the factors among the types of health information, types of posts, and times of post that are associated with good engagement rates among internet users. Whereby, an Internet user is identified as a user of the Internet who is involved in online communities or a mobile web user [21]. The findings of this study may facilitate health organizations in Malaysia and globally to improve their health communication strategies on social media and encourage health authorities to connect with their online audiences by increasing the creation of high-impact posts.

## 2. Materials and Methods 

### 2.1. Data Collection

Data was collected from the MOH FB page which is an official social media platform for our national health agency and is supervised by the Corporate Communication Unit (CCU) under a directive from the Secretary General’s Office. The MOH FB page is the most influential (in terms of total numbers of fans) among all health organization FB pages in Malaysia, according to Socialbakers.com, an artificial intelligence (AI)-powered social media analysis platform [22]. The FB page can be accessed through this link: www.facebook.com/kementeriankesihatanmalaysia. The data for this study was collected from FB Insight (an internal analysis tool provided by Facebook for self-evaluation) [23]. A total of 2899 FB posts were collected from the FB Insight. The data were exported into MS Excel format and identified by code numbers. All information collected was based on a proforma checklist including required variables such as the type of post, time of post, total likes, comments and shares of a post, and the number of internet users reached by a post. 

### 2.2. Inclusion Criteria

All FB posts in the form of a photo, video, link shared, or text on the MOH FB page dated between 1 November 2016 and 31 October 2017 (end of data collection) were included in this study.

### 2.3. Exclusion Criteria

Events, polls, app posts, and posts not originating from the MOH FB page were excluded from this study. The information written in other languages than Bahasa or English were excluded. 

### 2.4. Sample Size and Sampling Method

For sample size calculation, the single proportion formula was applied to estimate the required sample size for our first objective and PS Power and Sample Size Calculation software version 3.0 by William D. Dupont and Walton D. Plummer, Vanderbilt University, USA was used to calculate our second objective [24]. Based on all estimations, samples of 2123 FB posts were required for this study. The samples of this study were selected via simple random sampling method using SPSS software version 24.0, Armonk, NY, IBM Corp, Statistical Package for the Social Sciences, [25].

### 2.5. Type of Health Information

According to the Australian Government in their “Privacy Act,” health information is defined as information related to health, including health service provided for individuals or communities [26]. In our study, the types of health information were categorized into health education, organizational promotion, and risk communication, based on previous similar studies [16,27,28,29]. Risk communication is an exchange of real-time information, advice, and opinions between experts and people facing threats to their health or economic or social well-being [4,30]. Health education is educational information or news articles on a range of health topics delivered to the public or professionals, while organizational promotion is content or messages that advertise or build the image of an organization [31]. 

We used few techniques to analyze the text and graphic of the FB content. First, we applied an interpretive approach to analyzing the post with graphics on the FB platform. Then we made a preliminary judgement based on the interpretive approach that could represent the type of health information for the FB post. Our interpretations of graphics were constructed around our operational definitions and consultation from the number of sources. Next, we applied the text formulae to determine the presence of linguistic markers in each of the FB posts. The text formulae were based on the literature reviews and consultation with social media expert in the ministry that provide the definitions of the type of health information to identify linguistic markers in the FB contents. The risk communication posts were coded based on the presence of fifteen predefined keywords in Bahasa Malaysia translated into English such as “press statement”, “press release”, “harm”, “risk”, “threat”, “uncertain”, “public report”, “concern”, “warning”, “issue”, “problem”, “dangerous”, “peril”, “latest situation” or “serious”. Meanwhile, the organization promotional posts were coded with also fifteen predefined keywords such as “visit”, “launching”, “officiating”, “ceremony”, “watch”, “program”, “carnival”, “celebration”, “attend”, “congratulation”, “meeting”, “talk”, “campaign”, “service” or “facility” etc. Similar for educational posts, we used fifteen predefined keywords such as “sign”, “symptom”, “tips”, “what is”, “guideline”, “screening”, “treatment”, “management”, “info”, “infographic”, “way”, “benefits”, “practice”, “lets” or “how to”. If we reached uncertainty to decide the type of health information, we implemented a proximity analysis to confirm the type of health information. For example, if risk communication keywords are higher than other type of health information keywords in an FB post, it will be categorized as a risk communication post and vice versa.

### 2.6. Types of Posts

Types of FB posts can be categorized into photo, video, text only (status), link, shared video, event, game, poll, or app [32]. Based on our literature review, we opted for high media richness posts, which were photo, shared video, and video post, and low media richness posts, which were text status and link post [16].

### 2.7. Time of Post

The time of post in this study was categorized into weekday post and weekend post. Based on the previous studies [7,33], the time of day a post was made was further sub-divided into early morning post (0000H–0759H), morning post (0800H–1159H), afternoon post (1200H–1759H), and evening post (1800H–2359H).

### 2.8. Engagement Rate

Engagement rate on social media is defined as total number of engaged users, or interactive behaviors (number of likes, comments, and shares of a post) divided by audience reach (the number of internet users who saw a FB post at least once) [34,35]. Facebook provides the metadata of interactive behaviors and audience reach through their FB Insight tool. A FB post with a good engagement rate is greater than or equal to one percent. Meanwhile, a post with an engagement rate lower than 0.5% is considered as a poor engagement rate and a post with engagement rate lies between one percent and 0.5% is considered as an average [5,36]. 

### 2.9. Statistical Analysis

Descriptive statistics were used to present our first objective. All categorical data were presented as frequencies (percentages), while numerical data were presented as mean (SD) or median (IQR) based on their normality distribution. For our second objective, which was associations between types of post, time of post, type of health information as independent variables, and good engagement rate as the dependent variable, the univariable analysis was calculated by Simple Logistic Regression (SLR), while the multivariable analysis was assessed through Multiple Logistic Regression (MLR). In this study, only variables with *p*-values less than 0.025 or clinically important factors were selected for further analysis.

The preliminary model was obtained after the Forward Logistic Regression and Backward Logistic Regression methods were applied. The preliminary model consisted of the type of health information. Type of post and time of post were selected after the variable “Day Posted” was removed through the selection methods. Our model had shown weak correlation matrices between variables and relatively small standard errors, and no multicollinearity existed in the model. Furthermore, all possible two-way interactions were checked between the independent variables and were found to be insignificant. Hence, the preliminary final model was acquired to proceed with model fitness. The model fitness was tested by the Hosmer–Lemeshow test, classification table, and area under the receiver operating characteristic (ROC) curve. Our model was considered to be fit as evidenced by the Hosmer–Lemeshow test, which was not significant, the classification table, which was 65.6%, and the area under the ROC curve result, which was 68.4% (*p*-value = 0.001). All statistical analyses were conducted in SPSS software version 24. 

### 2.10. Ethics Approval 

An ethical clearance approval was obtained from the Human Research and Ethics Committee, Universiti Sains Malaysia (code USM/JEPeM/17120718) and the Medical Review and Ethical Committee (MREC) of the National Institutes of Health, Ministry of Health, Malaysia (code NMRR-17-3018-39127 [IIR]).

## 3. Results

Overall, organizational promotion (*n* = 766, 36.1%) was the most common type of health information posted, followed by health education posts (*n* = 707, 33.3%) and risk communication posts (*n* = 650, 30.6%). Most of the FB posts were posted during weekdays (*n* = 1669, 78.6%), and the remaining posts were posted during weekends (*n* = 454, 21.4%). The majority of posts in a day were posted between midnight and early morning, or before office hours (0000H to 0759H) (*n* = 870, 41.0%). The second most frequent posting time was during the evening after office hours (1800H to 2359H) (*n* = 799, 37.6%), followed by afternoon (1200H to 1759H) (*n* = 324, 15.3%), while the least frequent posting time was morning (*n* = 130, 6.1%) (0800H to 1159H). Amongst types of posts, photos (*n* = 1366, 64.3%) carried the highest proportion on the MOH FB page, followed by links (*n* = 533, 25.1%), shared videos (*n* = 133, 6.3%), and videos (*n* = 55, 2.6%), with text status being the least prevalent post type (*n* = 36, 1.7%). On average, the MOH posted six FB posts per day. Table 1 displays an overview of the post characteristics analyzed in this study.

Engagement with each post on social media is essential and one of the indicators of the effectiveness of health promotion. In our study, more FB posts on the MOH FB page showed average and poor engagement rates (*n* = 1291, 60.8%) than good engagement rates (*n* = 832, 39.2%). The mean of average and poor engagement rate was 0.54 (0.23) compared with the mean of good engagement rate, which was 1.82 (1.48). The formula for calculating the mean of good engagement rate was total good engagement rate of FB posts divided by the number of posts with good engagement rates, and a similar calculation was utilized for the mean of poor and average engagement posts. The findings are summarized in Table 2. 

The next objective of this study was to assess factors associated with engagement among internet users on FB. There was significant positive engagement with educational posts (Adjusted Odd Ratio (AOR): 3.80, 95% Confidence Interval (CI): 3.02–4.78, *p* < 0.001), risk communication posts (AOR: 1.77, 95% CI: 1.39–2.26, *p* < 0.001), video posts (AOR: 3.74, 95% CI: 1.44–9.71, *p* = 0.007), afternoon posts (AOR: 1.76, 95% CI: 1.34–2.31, *p* < 0.001), and posts made after office hours (AOR: 1.48, 95% CI: 1.20–1.82, *p* < 0.001). 

The coefficients of the logistic regression in our model indicate how the factors relate to good engagement rates. These results suggest that a health educational post has 3.8 odds of getting a good engagement rate with all other variables remaining constant, while a risk communication post has 1.77 odds of getting a good engagement rate. Of the various post types, only a video post increases the odds of getting a good engagement rate with odd ratio of 3.74, while in terms of time of post, afternoon and evening posts give higher odds of getting a good engagement rate with odd ratio of 1.76 and 1.48 respectively. 

However, other factors were not significantly associated with a good engagement rate. This study found that engagement was negatively associated with morning posts (AOR: 1.08, 95% CI: 0.72–1.62, *p* = 0.71), posts with links (AOR: 2.11, 95% CI: 0.96–4.66, *p* = 0.07), posts with shared videos (AOR: 0.67, 95% CI: 0.28–1.60, *p* = 0.36), and posts with photo elements (AOR: 1.82, 95% CI: 0.83–3.98, *p* = 0.13). Table 3 displays associations between type of health information, type of post, time of post, and good engagement rate using MLR. Together, these results provide important insights into the utilization of social media by an official health organization in Malaysia and its effectiveness in delivering health messages to internet users.

## 4. Discussion

The primary challenges in creating an interactive and engaging social media environment are the need to understand the dynamics of the FB algorithm to utilize a suitable type of post, recognizing audience behaviors to disseminate health messages at times most likely to achieve good engagement rates, and to consistently post appealing health content relevant to the audience to achieve good engagement rates [1,37,38]. 

Our study found that organizational promotion was the most prevalent type of health information posted on the MOH FB page, followed by health education posts and risk communication posts. This finding was similar to that of earlier studies on different types of information shared by health organizations on their social media accounts [31,39]. The finding also indicated that the MOH of Malaysia was not exploiting their social media interactive channel optimally. There is a need for a discussion regarding the viability and necessity of health organizations in Malaysia utilizing social media to improve public health activities. Published literature and local surveys have discovered increasing trends of online health-seeking behavior among the public [11,40,41]. As demands for online health information are increasing rapidly, health organizations should optimize their social media accounts and provide more evidence-based health information to internet users. One of the essential public health services for a health organization as suggested by CDC is to inform, educate and empower the public on health issues and using online platforms is an effective way to meet that goal [42].

Another important finding was that the photo and the link emerged as the most frequent post types utilized on the MOH FB page. This finding was similar to that of several studies on FB pages of several health organizations in Australia [32] and a CDC FB page analysis [43]. However, other studies have found that text status updates or links were more commonly utilized on FB pages than high media-rich posts [2,29]. A possible explanation for this might be that some health organizations prefer internet users to consult health information on their health portals, with FB acting as a funnel to direct internet users to the websites. Additionally, preferences for low media-rich post types may be related to their technical simplicity and the lower level of IT knowledge required by FB administrators to create a FB post. Following the present results, a previous study has demonstrated that the most common time for posts was the early morning, followed by the evening after office hours [43]. However, a study on a pharmacy organization in Canada has shown a different finding. That study found that the most common posting times on the pharmacy FB page in 2013 were the morning and afternoon. A year later, the highest number of posts was seen during the evening period. The difference in findings resulted from the pharmacy’s strategy to increase the frequency of evening posts in 2014 compared with 2013 to achieve greater audience reach [33].

One interesting finding was that more FB posts on the MOH FB page achieved average and poor engagement rates than good engagement rates. Social media engagement is one of the indicators for the effectiveness of health communication [1]. This result may be a signal to the MOH to galvanize their effort to capture the attention of internet users and influence their perception and behaviors through high-impact health messages [32,43]. 

Our analysis also acknowledged some factors that may augment the diffusion of health content by increasing user engagement. This study discovered strong relationships between health education posts and risk communication posts with good engagement rates. Consistent with the literature, this research found a similar result to several studies done in developed countries such as Canada, the U.S., and Australia [7,32,44]. Surprisingly, video was the only type of post found to have a significant association with good engagement rates compared with other post types. The finding was supported by an analysis of FB pages in Australia [32]. Another important finding was that FB posts delivered in the afternoon (1200H–1759H) and evening (1800H–2359H) showed significant associations with good engagement rates among internet users, and the finding was also supported by some earlier studies [33,43].

Several reports have shown that many health organizations are still focusing on “pushing” information such as organizational promotion posts to internet users, rather than encouraging participation and engagement-related content on social media [17,31]. A study of health agencies’ participation on social media revealed organizational promotion posts associated with low engagement rates among internet users. This relationship may be partly explained by the circumstance that such posts are focused more on organization interest than public interest [2]. One of the methods to improve engagement is to provide relevant educational and news content for the audience [17]. Risk-related information appealing to emotions, particularly fear and curiosity, can change public perception and stimulate a positive response [45,46].

Moreover, FB posts that have been based on the social amplification of risk framework (SARF) pivoted on the volume of risk information, the vagueness of information, dramatization of information, and essential symbolic connotations, have been predicted to improve public engagement [47]. Nevertheless, health organizations must share risk communication posts judiciously, as they may influence viewers’ evaluations and stimulate negative interactions such as public panic, which may affect the engagement rate and create unnecessary public health threats [19]. Conversely, similar to risk information, an educational post will enhance a population’s health literacy, fostering social support, reducing stigmas and myths about many health issues, and improving personal health management [48,49].

Meanwhile, there were conflicts of results and frameworks for anticipating the best type of post to achieve better engagement. Previous studies have noted the importance of the Common-Sense Model application in FB posts with imagery elements to encourage greater audience engagement [2,44,46]. However, a study on online engagement factors found that a video post using The Laugh Model framework compared with other media-richness theories or frameworks was able to achieve an engagement rate of up to 10%, whereas more than 1% is already considered a good engagement rate [5]. Another content analysis of the FB page of a specific target group in Canada has predicted that good engagement rate is correlated with link, video, or photo posts [7]. Comparison of the findings between previous studies and ours confirmed the effect of a new machine-learning FB algorithm that replaced the previous Edge Rank algorithm. The new FB algorithm prioritizes video posts to appear on news feeds more frequently than other post types [50]. Moreover, Cisco has projected that video traffic will account for more than 80% of all Internet consumption in the next three years globally [51]. Although video has better media richness compared with photo according to the media richness theory [16], it is not the only factor that garners a good engagement rate. Other factors, such as sentiments and semantics relevant to the audience with mobile-friendly features, may influence the engagement rate of a video post [44].

One theory has suggested that a FB post made during an active period resembling working hours will achieve a significant engagement rate [7]. However, there were mixed findings from previous studies to support the hypothesis [7,33,43]. A possible explanation for these results may be the restriction of social media usage in the workplace and slow Internet connections due to massive Internet access rates during office hours in Malaysia [41]. These factors have contributed to the low prevalence of morning posts and poor engagement rates during office hours. One of the solutions to counter these obstacles is to apply social media assistance software with AI. The high technology software is capable of predicting online audience behaviors and can timely disseminate high-impact messages during more active periods [7,33].

These findings should be interpreted with some consideration of the limitations of this study. One of the issues that emerged from the study is that the MOH FB page was not selected randomly from the social media accounts of all health organizations in Malaysia using a randomized approach. It is difficult to say whether the study’s findings are suitable to be generalized to all health agencies in Malaysia. However, the MOH FB page is the most influential health-related FB page in Malaysia according to Socialbakers.com [22]. Therefore, it is possible that our results can be taken to represent all health organizations in Malaysia. Second, due to time feasibility and limited resources, this study applied a cross-sectional model to establish associations between variables and outcomes. In the future, conducting an experimental study or cohort study with adequate resources may establish the causal pathway. Third, social media algorithms are aggressive and dynamic. We speculated that the FB algorithm would continuously evolve to enhance user experience and will alter engagement factors on FB in the future. Therefore, periodic analysis of engagement factors on social media, especially after a change to a new algorithm, is necessary to establish effective strategies to engage with internet users for specific time periods. Finally, social media is one of the tools for conveying health messages to the public. Although it is undisputedly able to reach a larger audience with better communication, engagement, and cost-effective levels compared with other conventional tools, we are not sure if the public truly understood the health messages and subsequently modified their behaviors in response. Therefore, further qualitative studies may be able to determine the effect of health communication via social media on changes in human knowledge, attitudes, and real-life perceptions

## 5. Conclusions

The growing trend of online health-seeking behaviors and demand for the availability of validated health information on social media platforms requires effective strategies by health organizations to disseminate health information to internet users. An educational or risk communication post appealing to emotions such as curiosity, fear, and anxiety can change public perception and encourage behavioral actions. Moreover, a FB post with video content and timely dissemination of messages can achieve better audience engagement on social media. These findings have important implications for the development of effective social media health communication strategies by health organizations in Malaysia. Although a causal conclusion cannot be inferred from this study, at a minimum, we can conclude that the health information factors we analyzed in this study will achieve better engagement rates on social media.

## Figures and Tables

**Table 1:** Characteristics of posts on the Ministry of Health Facebook (MOH FB) page (*n* = 2123).

Variables		*n* (%)
Type of Health Information		
	Organizational Promotion	766 (36.1)
	Health Education	707 (33.3)
	Risk Communication	650 (30.6)
Day Posted		
	Weekend	454 (21.4)
	Weekday	1669 (78.6)
Time Posted		
	0000H–0759H	870 (41.0)
	0800H–1159H	130 (6.1)
	1200H–1759H	324 (15.3)
	1800H–2359H	799 (37.6)
Type of Post		
	Status	36 (1.7)
	Link	533 (25.1)
	Shared Video	133 (6.3)
	Video	55 (2.6)
	Photo	1366 (64.3)

**Table 2:** Engagement rates of posts on MOH FB page (*n* = 2123).

Variable		*n* (%)	Mean (SD) of Engagement Rate
Engagement Rate	Average and Poor	1291(60.8)	0.54 (0.23)
	Good	832 (39.2)	1.82 (1.48)

**Table 3:** Association between type of health information, type of post, time of post, and good engagement rate using MLR.

Variables		Crude OR (95% CI) ^a^	Adjusted OR (95% CI) ^b^	*p*-Value ^b^
Type of Health Information				
	Organizational Promotion	1	1	
	Health Education	3.79 (3.04–4.73)	3.80 (3.02–4.78)	<0.001
	Risk Communication	1.88 (1.50–2.36)	1.77 (1.39–2.26)	<0.001
Time Posted				
	0000H–0759H	1	1	
	0800H–1159H	1.12 (0.76–1.65)	1.08 (0.72–1.62)	0.71
	1200H–1759H	2.08 (1.60–2.70)	1.76 (1.34–2.26)	<0.001
	1800H–2359H	1.51 (1.23–1.84)	1.48 (1.20–1.82)	<0.001
Type of Post				
	Status	1	1	
	Link	2.37 (1.09–5.13)	2.11 (0.96–4.66)	0.07
	Shared Video	0.99 (0.42–2.32)	0.67 (0.28–1.60)	0.36
	Video	4.17 (1.66–10.53)	3.74 (1.44–9.71)	0.007
	Photo	1.86 (0.87–3.99)	1.82 (0.83–3.98)	0.13

^a^ Simple Logistic Regression; ^b^ Multiple Logistic Regression (MLR): Constant = −1.923; Forward Logistic Regression (LR) and Backward LR were applied. No Multicollinearity and Interaction detected; Hosmer–Lemeshow Test, *p*-value = 0.73; Classification table was 65.6% correctly classified. Area under receiver operating characteristic curve was 68.4%. OR: odd ratio; CI: confidence interval.
